# Dietary L-arginine supplementation exerts preventive effects on colitis through modulation of the gut microbiota

**DOI:** 10.3389/fnut.2026.1848380

**Published:** 2026-05-29

**Authors:** Lerun Gong, Jing Kong, Xiaoying Shao, Weiming Meng, Ruxin Zhang, Yanxi Zhang, Yu Feng

**Affiliations:** 1 School of Life Science and Engineering, Jining University, Jining, China; 2 The Clinical College, Chengde Medical University, Chengde, China

**Keywords:** gut microbiota, L-arginine, preventive, therapeutic, ulcerative colitis

## Abstract

**Background:**

Ulcerative colitis (UC) is a chronic, relapsing gastrointestinal disease that imposes an increasing global health burden. Our previous study showed that L-arginine (Arg) could markedly alleviate experimental colitis; however, the relative efficacy of its prophylactic versus therapeutic intervention remains unclear, and the underlying mechanisms require further elucidation.

**Methods:**

Mice received Arg supplementation either prior to DSS exposure (BeArg) or during DSS treatment (DuArg). Intestinal barrier function, inflammatory cytokines and gut microbiota alterations were evaluated, followed by fecal microbiota transplantation (FMT) validation.

**Results:**

BeArg markedly alleviated DSS-induced mice body weight loss, disease activity index (DAI) elevation, and colon shortening, exhibiting a protective efficacy comparable to full-course Arg administration. BeArg also lowered serum lipopolysaccharide, consistent with improved intestinal barrier integrity. In addition, BeArg reduced the expression of tumor necrosis factor-alpha (TNF-α) and interferon-gamma (IFN-γ) while augmenting interleukin-10 (IL-10) at both the transcriptional and protein levels. By comparison, DuArg produced only modest clinical improvement and showed limited efficacy in modulating barrier dysfunction and inflammatory responses. 16S rRNA sequencing revealed that BeArg and Arg interventions induced similar alterations in the gut microbial community structure, while FMT further confirmed that Arg-mediated remodeling of the gut microbiota effectively protected against DSS-induced colitis.

**Conclusion:**

These data indicate that Arg exerts a prophylactic effect against colitis by modulating the gut microbiota, underscoring the pivotal role of intervention timing in optimizing its protective effects.

## 1 Introduction

Ulcerative colitis (UC) is a chronic, relapsing gastrointestinal disease characterized by continuous injury and inflammation of the colonic mucosa and submucosa (1). Clinically, patients typically present with hematochezia, persistent diarrhea, and abdominal pain. Moreover, long-standing and/or extensive disease is associated with an increased risk of colorectal cancer (2). Epidemiological evidence indicates that the incidence and prevalence of UC are rising globally, posing a serious threat to public health (3, 4). Current pharmacological therapies, mainly including aminosalicylates, systemic corticosteroids, and immunosuppressants, remain the main treatment options for UC; however, their clinical utility is constrained by limited efficacy, high cost, and potential adverse effects (5). Thus, there is an urgent need to develop novel, safe, and effective therapeutic strategies for UC.

Although the precise pathogenesis of UC remains incompletely elucidated, gut microbiota dysbiosis and impairment of the intestinal barrier are widely recognized as central contributors to its initiation and progression (6–8). The gut microbiota, consisting of trillions of microorganisms that inhabit the gastrointestinal tract, plays an essential role in maintaining intestinal homeostasis through the regulation of nutrient metabolism, immune responses, and host defense. Accumulating evidence indicates that UC is associated with profound alterations in gut microbial composition, characterized by the depletion of commensals associated with intestinal homeostasis and the expansion of bacteria enriched in inflammatory conditions (9, 10). For example, the abundance of butyrate-producing bacteria (e.g., *Faecalibacterium prausnitzii* and *Roseburia*) decreases, whereas potentially pro-inflammatory bacteria including *Escherichia coli* and *Shigella* are enriched. Experimental studies further support a causal role for the gut microbiota in colitis. Schaubeck et al. (11) showed that transplantation of fecal microbiota from colitis mice into healthy recipients precipitated colitic pathology, whereas transfer of microbiota derived from healthy human donors into experimentally colitis mice markedly ameliorated the inflammatory milieu. Collectively, these findings suggest that gut microbiota dysbiosis contributes to the disruption of epithelial barrier integrity and mucosal immune homeostasis, thereby promoting a self-perpetuating cycle of chronic inflammation. Given that the gut microbiota is highly responsive to dietary factors, dietary interventions aimed at modulating the intestinal microbial ecosystem might represent a promising strategy for the prevention and treatment of UC.

Arg is a semi-essential amino acid that plays a central role in mammalian nitrogen metabolism and cellular signaling. Arg is derived endogenously from citrulline and exogenously from dietary intake. It serves as the obligate substrate for nitric oxide synthases (NOS), which catalyze the production of nitric oxide (NO) and citrulline, and is also metabolized by arginase to produce ornithine and urea, thereby supporting the biosynthesis of polyamines and proline required for cell proliferation and tissue repair (12–14). In addition to its established clinical applications in hyperammonemia-associated hepatic encephalopathy and male infertility, Arg has attracted increasing attention for its potential role in intestinal health. Wei et al. (15) found that Arg could downregulate pro-inflammatory cytokines and reduce intestinal permeability, yielding significant outcomes in alleviating the symptoms of UC. Antonio et al. (16) discovered that the remodeling of Arg metabolism could mitigate gut barrier dysfunction. We have likewise demonstrated that dietary Arg supplementation significantly improves DSS-induced colitis in mice (17). Despite these encouraging findings, it remains unclear whether the anti-colitic effects of Arg are predominantly prophylactic or therapeutic, and the underlying mechanisms have yet to be fully elucidated. To clarify this issue, the present study employs dextran sulfate sodium (DSS) to induce experimental colitis and systematically compared the efficacy of Arg supplementation initiated before versus following disease induction. The findings of this study might help to define the optimal timing of Arg intervention and provide a basis for the development of nutrition-based strategies for the prevention and management of UC.

## 2 Materials and methods

### 2.1 Animal experiment

**Figure 1:**
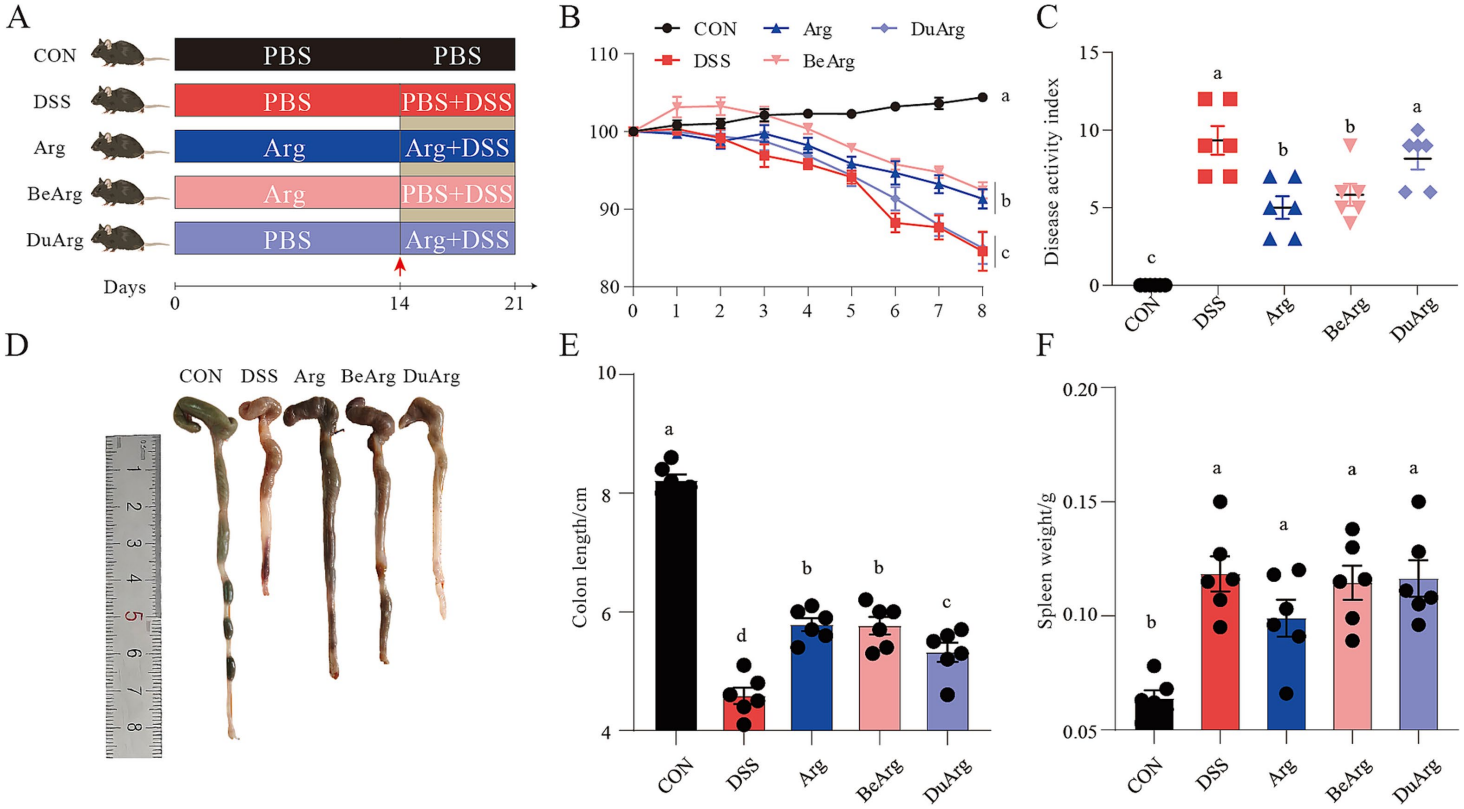
Effects of BeArg and DuArg on DSS-induced colitis in mice. **(A)** Experimental design; **(B)** Body weight loss; **(C)** DAI score; **(D)** Representative macroscopic images of colon tissues from each experimental group; **(E)** Colon length; **(F)** Spleen weight. *n* = 6. Groups labeled with different letters are significantly different (*p* < 0.05).

The animals used in this experiment were purchased from Beijing Huafukang Biotechnology Co., Ltd. As illustrated in Figure 1A, after a 7-day acclimatization, 4-5-week-old male C57BL/6 J mice were randomized into 5 groups (*n* = 6 per group): CON, DSS, Arg, BeArg, and DuArg. Arg was administered as a solution by oral gavage at a dose of 2 g·kg^−1^ body weight for the designated treatment intervals. The Arg group received Arg for the entire experimental period. The BeArg group was given Arg prior to DSS exposure, whereas the DuArg group received Arg concomitant with DSS administration. CON and DSS groups were gavaged with an equivalent volume of PBS. In the present study, exclusively male mice were utilized to minimize inter-individual variability and establish a stable baseline for microbial and immunological evaluations. This choice was made to eliminate the confounding effects of the female estrous cycle and fluctuating sex hormones, notably estrogen. Animal experiments were conducted following the National Institutes of Health guide for the care and use of Laboratory Animals (NIH Publications No. 8023, revised 1978), and the protocols were reviewed and approved by the Animal Care and Ethics Committee of Jining University (Ethics reference number: 2025JNXYLL-025).

### 2.2 DAI

DAI was used to quantify the severity of intestinal inflammation. It was calculated based on three parameters (Supplementary Table S1), with a maximum total score of 12.

### 2.3 Histological assessment

Colonic tissues were fixed in 4% paraformaldehyde, embedded in paraffin, sectioned (4–5 μm), and subjected to hematoxylin and eosin (H&E) staining. Histological injury was evaluated according to the criteria shown in Supplementary Table S2, as previously described (6).

Goblet cells were visualized by Alcian blue (AB) staining using a commercial kit (Servicebio Technology Co., Ltd., Wuhan, China). Quantification was conducted with ImageJ software.

### 2.4 Inflammatory cytokine detection

Colonic tissue levels of inflammatory cytokines (TNF-α, IFN-γ, and IL-10) were measured using enzyme-linked immunosorbent assay (ELISA) and RT-qPCR. ELISA assays were performed according to the manufacturer’s instructions, and cytokine concentrations were calculated from standard curves and normalized to sample volume or tissue protein content.

### 2.5 Quantitative reverse transcription polymerase chain reaction (RT-qPCR)

Total RNA was extracted from colon tissues using the Tiangen RNA extraction kit (Tiangen Biotech, Beijing, China) according to the manufacturer’s instructions. Complementary DNA (cDNA) was synthesized using a reverse transcription kit. Quantitative real-time PCR (RT-qPCR) was performed to assess the expression levels of target genes, with primers listed in the Supplementary Table S3. Relative gene expression was calculated using the 2^−ΔΔCt^ method.

### 2.6 16S rRNA sequence

Fresh fecal samples were collected, and genomic DNA was extracted using a commercial kit according to the manufacturer’s instructions. The V3-V4 region of the bacterial 16S rRNA gene was amplified using primers 338F/806R and sequenced on an Illumina NextSeq2000 platform. Raw paired-end reads were quality-filtered with fastp, merged using FLASH, and denoised using the DADA2 plugin in QIIME2 to generate amplicon sequence variants (ASVs). Chloroplast and mitochondrial sequences were removed. To control for differences in sequencing depth, all samples were rarefied to 20,000 reads per sample, yielding an average Good’s coverage of 97.9%. Microbial community composition and shared taxa were visualized using Venn analysis. Alpha diversity (Chao1, Shannon) and beta diversity (PCoA based on Bray-Curtis distances) were calculated, with group differences evaluated using the Wilcoxon rank-sum test and PERMANOVA, respectively. Differentially abundant taxa were identified using LEfSe with an LDA score cutoff >2 and *p* < 0.05, and *p*-values were corrected for multiple comparisons using the Benjamini-Hochberg false discovery rate. The sequencing data have been deposited in the NCBI SRA under accession number PRJNA1439605.

### 2.7 FMT

To deplete the resident gut microbiota, recipient mice were administered a broad-spectrum antibiotic cocktail for 2 weeks before FMT. In parallel, donor mice in the Arg group were treated with Arg for 2 weeks, whereas donor mice in the control group received an equal volume of PBS. Fresh fecal samples were collected from donor mice and immediately homogenized in sterile PBS. The fecal suspensions were orally administered to recipient mice in the PBS recipient and Arg recipient groups for 2 consecutive weeks. Following FMT, colitis was induced in recipient mice by 3% DSS drinking water treatment.

### 2.8 Statistical analysis

Data are presented as Mean ± SEM. Differences between groups were evaluated using one-way analysis of variance (ANOVA) followed by Tukey’s multiple comparisons *post hoc* test. Statistical analyses were performed using GraphPad Prism 9, and *p* < 0.05 was considered statistically significant.

## 3 Results

### 3.1 Effects of BeArg and DuArg on DSS-induced colitis in mice

As shown in Figure 1, DSS administration induced typical colitis manifestations, including significant body-weight loss, increased disease activity index (DAI), marked colon shortening, and splenomegaly. Consistent with our previous results (17), continuous Arg supplementation throughout the experimental period (Arg group) markedly mitigated colitis (Figures 1B–F). Intriguingly, both BeArg and Arg showed comparable protective effects, significantly reducing weight loss and DAI, and preserving colon length and macroscopic colonic morphology. In contrast, DuArg only modestly improved colon length, without significantly affecting DAI and weight loss (Figures 1B–F). These results indicate that Arg might primarily exert its anti-inflammatory effects in a preventive manner.

### 3.2 Effects of BeArg and DuArg on the pathology of mouse colon tissue

**Figure 2:**
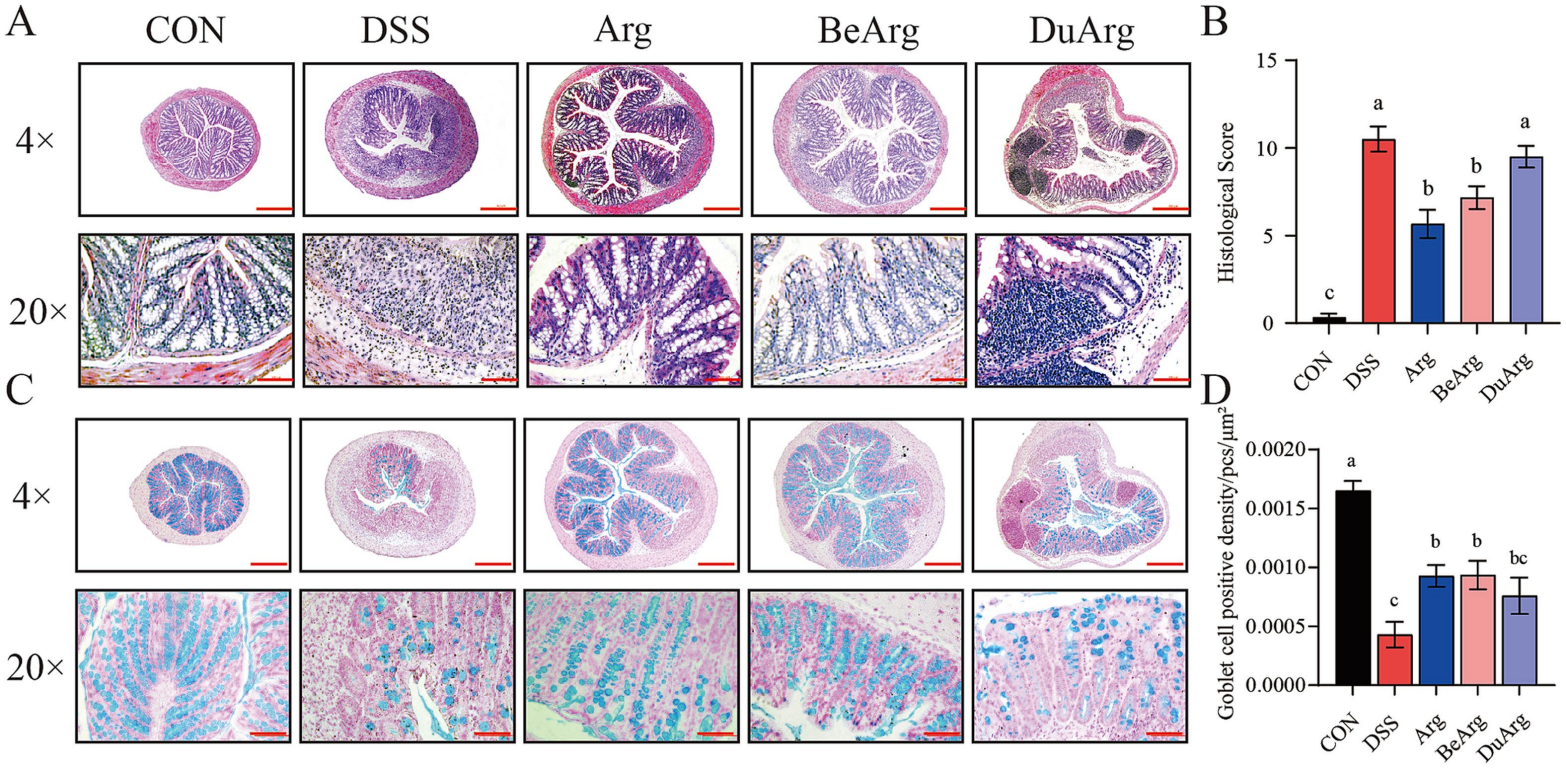
Effects of BeArg and DuArg on the pathology of mouse colon tissue. **(A)** Representative H&E-stained section images. 4 × scale bar, 500 μm; 20 × scale bar, 100 μm; **(B)** Histological score; **(C)** Representative AB stained section images. 4 × scale bar, 500 μm; 20 × scale bar, 100 μm; **(D)** Goblet cell positive density. *n* = 6. Groups labeled with different letters are significantly different (*p* < 0.05).

To further evaluate the effects of Arg intervention on improving colitis at different time points, we conducted a histological examination of the intestinal tissue. As depicted in Figure 2A, compared to the CON group, DSS administration induced pronounced colonic mucosal ulceration and erosion, accompanied by dense inflammatory cell infiltration and a marked disruption of the crypt architecture. However, Arg intervention could significantly ameliorate the pathological damage induced by DSS (Figures 2A,B). Specifically, the BeArg group, in which Arg was administered prior to the induction of colitis, exhibited symptom improvements comparable to those observed in the Arg intervention group. The DuArg group showed a trend toward improvement, although no significant difference was found when compared to the DSS group. Consistent with the H&E staining results, AB staining demonstrated that DSS treatment significantly reduced the number of goblet cells in the intestinal tissue compared with the CON group. Both BeArg and Arg interventions markedly alleviated DSS-induced goblet cell depletion, with comparable efficacy observed between the two groups. In contrast, the DuArg intervention did not result in a significant difference relative to the DSS group (Figures 2C,D).

### 3.3 Effects of BeArg and DuArg on intestinal barrier integrity in colitis mice

**Figure 3:**
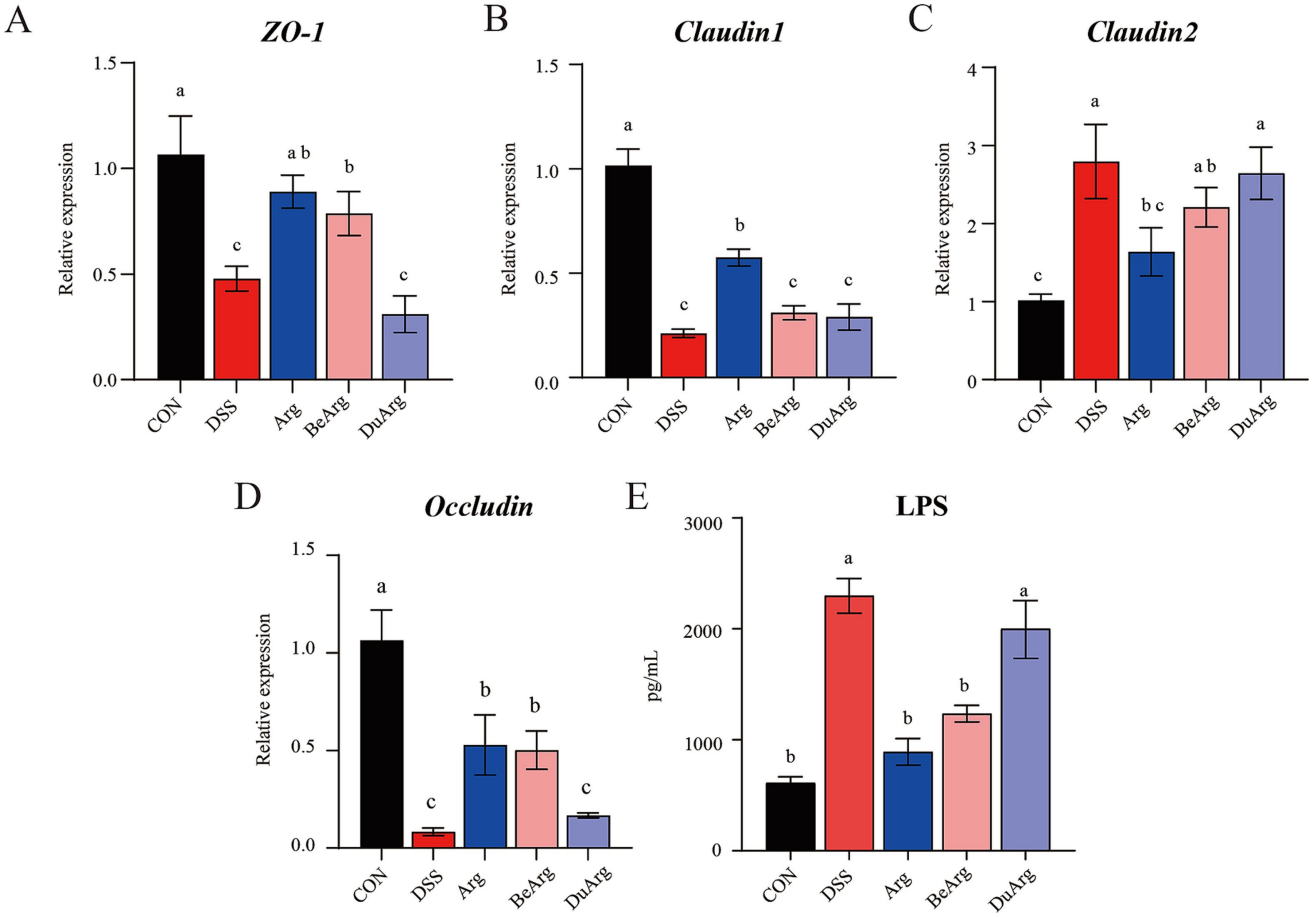
Effects of BeArg and DuArg on intestinal barrier integrity in colitis mice. **(A–D)** The relative expression levels of *ZO-1*, *Claudin-1*, *Claudin-2*, and *Occludin* genes. **(E)** Serum LPS levels. *n* = 6. Groups labeled with different letters are significantly different (*p* < 0.05).

The intestinal epithelial barrier is a physical structure formed by tight junctions between adjacent intestinal epithelial cells, which separates luminal contents from the internal environment and plays a critical role in maintaining host health. RT-qPCR analysis demonstrated that, compared with the CON group, DSS treatment significantly downregulated the mRNA expression of the tight junction-associated genes *ZO-1*, *Claudin-1*, and *Occludin*, while markedly upregulating *Claudin-2* expression, indicating disruption of intestinal barrier integrity. BeArg intervention significantly reversed these DSS-induced alterations in tight junction gene expression, exhibiting effects comparable to those observed in the Arg group. In contrast, the effects of DuArg intervention were significantly weaker and differed markedly from those of Arg intervention (Figures 3A–D). Consistent with these findings, serum lipopolysaccharide (LPS) levels, an established indicator of intestinal barrier dysfunction, were significantly elevated following DSS treatment. However, both Arg and BeArg interventions markedly reduced serum LPS concentrations, further supporting their protective roles in maintaining intestinal barrier integrity (Figure 3E).

### 3.4 Effects of BeArg and DuArg on inflammatory cytokine expression in colitis mice

**Figure 4:**
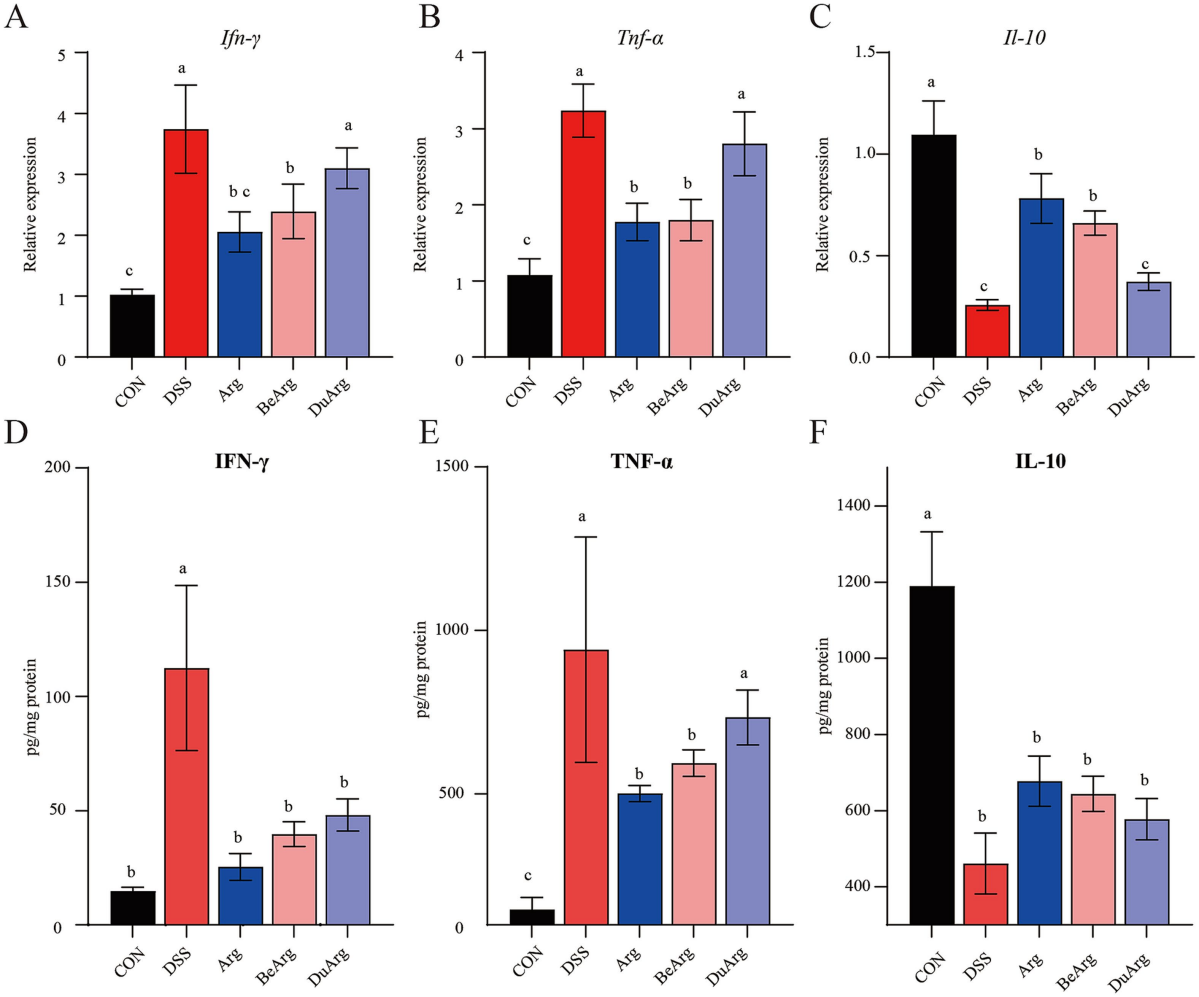
Effects of BeArg and DuArg on inflammatory cytokine expression in colitis mice. **(A–C)** Relative expression of IFN-γ, TNF-α, and IL-10 genes in colon tissue; **(D–F)** Content of IFN-γ, TNF-α, and IL-10 in colon tissue. *n* = 6. Groups labeled with different letters are significantly different (*p* < 0.05).

Inflammatory cytokines are key mediators of the initiation and progression of UC. Consistent with this, DSS treatment significantly elevated the expression of the proinflammatory cytokines *Ifn-γ* and TNF-α in colonic tissue, while markedly reducing the level of the anti-inflammatory cytokine *Il-10* (Figures 4A–C). Both BeArg and Arg interventions effectively restored the DSS-induced cytokine imbalance, exhibiting comparable efficacy. By contrast, DuArg treatment only partially ameliorated the abnormal cytokine profile, with no statistically significant differences observed relative to the DSS group. To further validate these findings at the protein level, we measured colonic cytokine concentrations by ELISA. Compared with the DSS group, BeArg and Arg interventions significantly improved the concentrations of IFN-*γ* (39.71 ± 5.41 vs. 112.40 ± 36.12 pg./mg protein, 25.39 ± 5.89 vs. 112.40 ± 36.12 pg./mg protein, respectively), TNF-α (594.39 ± 40.56 vs. 941.22 ± 344.80 pg./mg protein, 501.71 ± 24.83 vs. 941.22 ± 344.80 pg./mg protein, respectively), and IL-10 (643.99 ± 46.41 vs. 460.92 ± 79.90 pg./mg protein, 577.27 ± 54.11 vs. 460.92 ± 79.90 pg./mg protein, respectively), whereas DuArg showed only a limited effect on these cytokines (Figures 4D–F).

### 3.5 Effects of BeArg and DuArg on gut microbiota composition and structure

**Figure 5:**
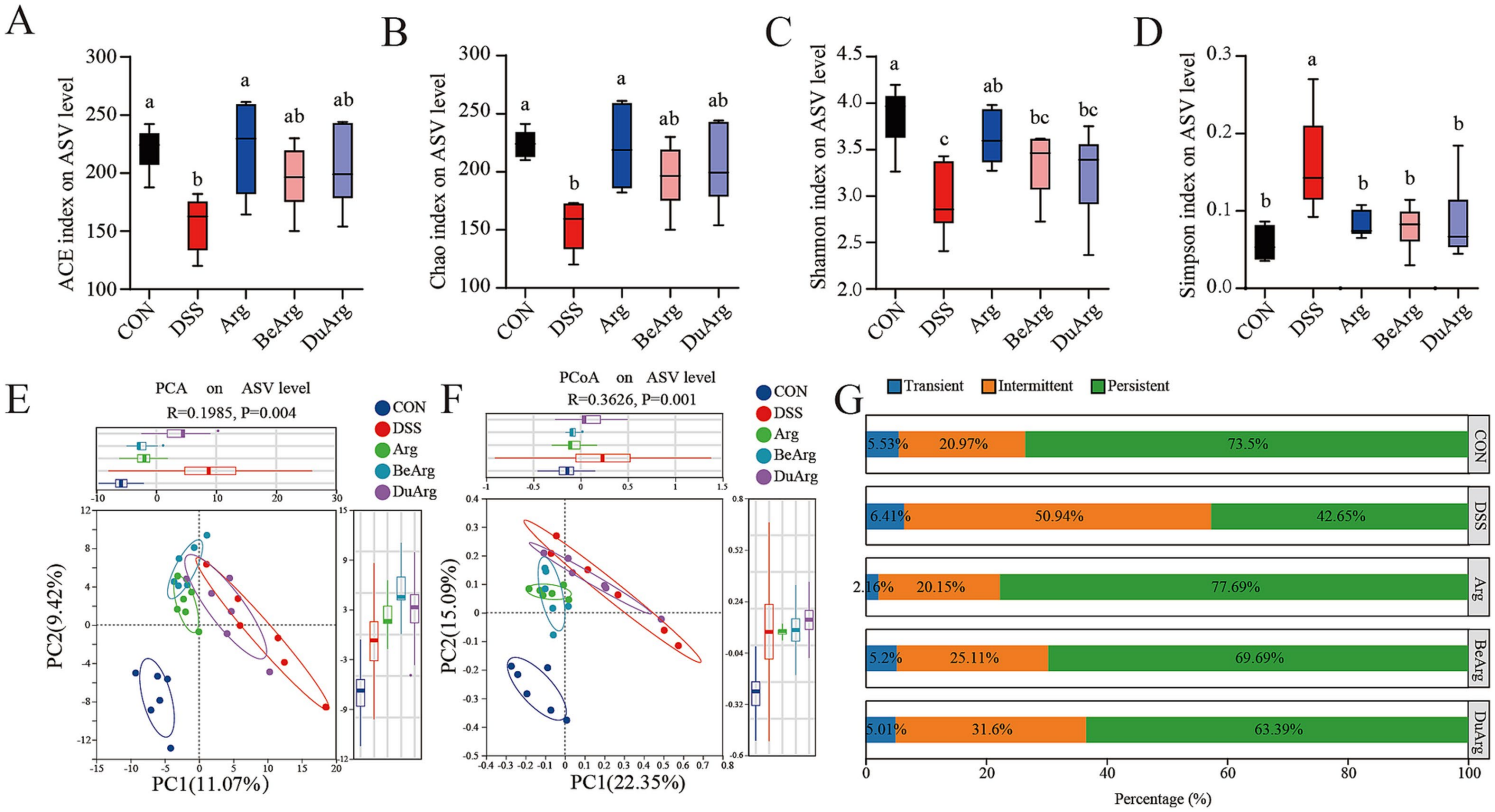
Effects of BeArg and DuArg on α diversity and *β* diversity of gut microbiota. **(A)** Ace index; **(B)** Chao index; **(C)** Shannon index; **(D)** Simpson index; **(E)** Principal component analysis of gut microbial communities in mice; **(F)** Principal coordinates analysis of gut microbial communities in mice. **(G)** Relative proportions of transient, persistent, and intermittent microbial taxa in each group. *n* = 6. Groups labeled with different letters are significantly different (*p* < 0.05).

Gut microbiota dysbiosis is considered a major contributor to the development and progression of UC. To elucidate the differences between the BeArg and DuArg interventions, 16S rRNA sequencing was employed to characterize the effects of these treatments on the composition of the gut microbiota. *α*-diversity, assessed by Chao1, ACE, Shannon, and Simpson indices, showed that DSS treatment significantly reduced microbial richness and diversity compared with the CON group. Both Arg and BeArg interventions effectively restored α-diversity across all four indices, whereas DuArg treatment resulted in only partial improvement without reaching statistical significance (Figures 5A–D). Principal component analysis (PCA) and principal coordinates analysis (PCoA) revealed distinct clustering patterns among the groups. DSS-treated mice formed a separate cluster from the CON group, indicating marked dysbiosis. The Arg and BeArg interventions shifted the microbial communities toward the healthy control cluster, whereas DuArg treatment produced only minimal improvement (Figures 5E,F). Moreover, *β* diversity analysis demonstrated that the Arg and BeArg groups exhibited similar microbial community structures, which may partially underlie the comparable effects of the BeArg and Arg interventions.

To further characterize the effects of these interventions on gut microbiota, we analyzed the distribution of transient, persistent, and intermittent microbial taxa, followed by a detailed taxonomic assessment at the phylum and genus levels. Ecological type analysis revealed that DSS treatment markedly altered the composition of gut microbiota, characterized by an increased proportion of intermittent taxa and a concomitant reduction in persistent taxa, indicating an unstable microbial ecosystem (Figure 5G). Both Arg and BeArg interventions significantly restored the proportion of persistent taxa while reducing transient taxa, suggesting an improvement in microbial stability.

**Figure 6:**
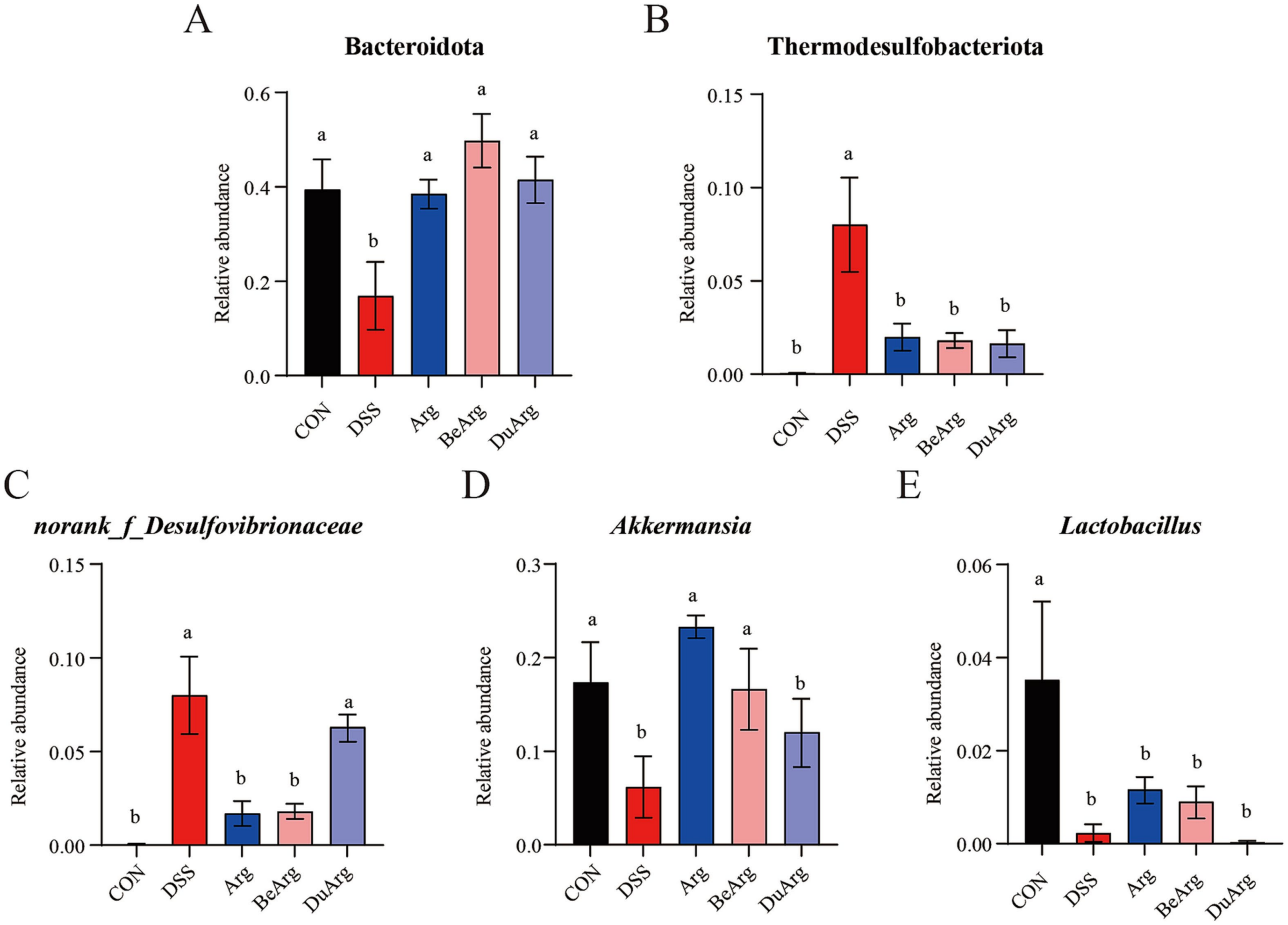
Alterations in gut microbial composition at phylum and genus levels. **(A,B)** Relative abundance of Bacteroidota and Thermodesulfobacteriota; **(C–E)** Relative abundance of *Desulfovibrio*naceae, *Akkermansia,* and *Lactobacillus*. *n* = 6. Groups labeled with different letters are significantly different (*p* < 0.05).

Building upon these observations, we next examined the taxonomic composition of gut microbiota. At the phylum level, DSS treatment resulted in a decreased relative abundance of Bacteroidota and an increased abundance of Thermodesulfobacteriota (Figures 6A,B; Supplementary Figure S1A). Arg and BeArg interventions effectively rebalanced these dominant phyla. At the genus level, DSS-induced dysbiosis was characterized by a reduction in genera associated with intestinal homeostasis, such as *Akkermansia* and *Lactobacillus*, accompanied by an enrichment of taxa enriched in inflammatory conditions, including *Desulfovibrio*naceae and *Lachnospiraceae_NK4A136_group* (Figures 6C–E; Supplementary Figure S1B). These alterations were substantially reversed by Arg and BeArg interventions, while DuArg showed only modest modulation. Collectively, these findings indicate that Arg and BeArg interventions not only improve the taxonomic composition of gut microbiota but also enhance microbial ecological stability, whereas DuArg exerts a comparatively weaker regulatory effect.

### 3.6 Key gut microbial biomarkers associated with BeArg and DuArg interventions

LEfSe analysis identified distinct microbial taxa associated with each experimental group (Supplementary Figures S2, S3). The CON group was enriched in beneficial taxa, including *Muribaculaceae* and *Lactobacillus*. In contrast, the DSS group showed significant enrichment of inflammation-associated taxa, such as *Desulfovibrionales* and *Helicobacter*. The Arg group was characterized by enrichment of taxa, including *Akkermansia*, *norank_f_Muribaculaceae*, and *Odoribacter*, suggesting partial restoration of gut microbial structure. Notably, the BeArg group exhibited a distinct enrichment of Bacteroidota-related taxa, while the DuArg group was mainly enriched in Alphaproteobacteria-associated taxa, such as *Rikenellaceae_RC9_gut_group* and *Rhodospirillales*.

### 3.7 Correlation between key taxa and inflammation indices

**Figure 7:**
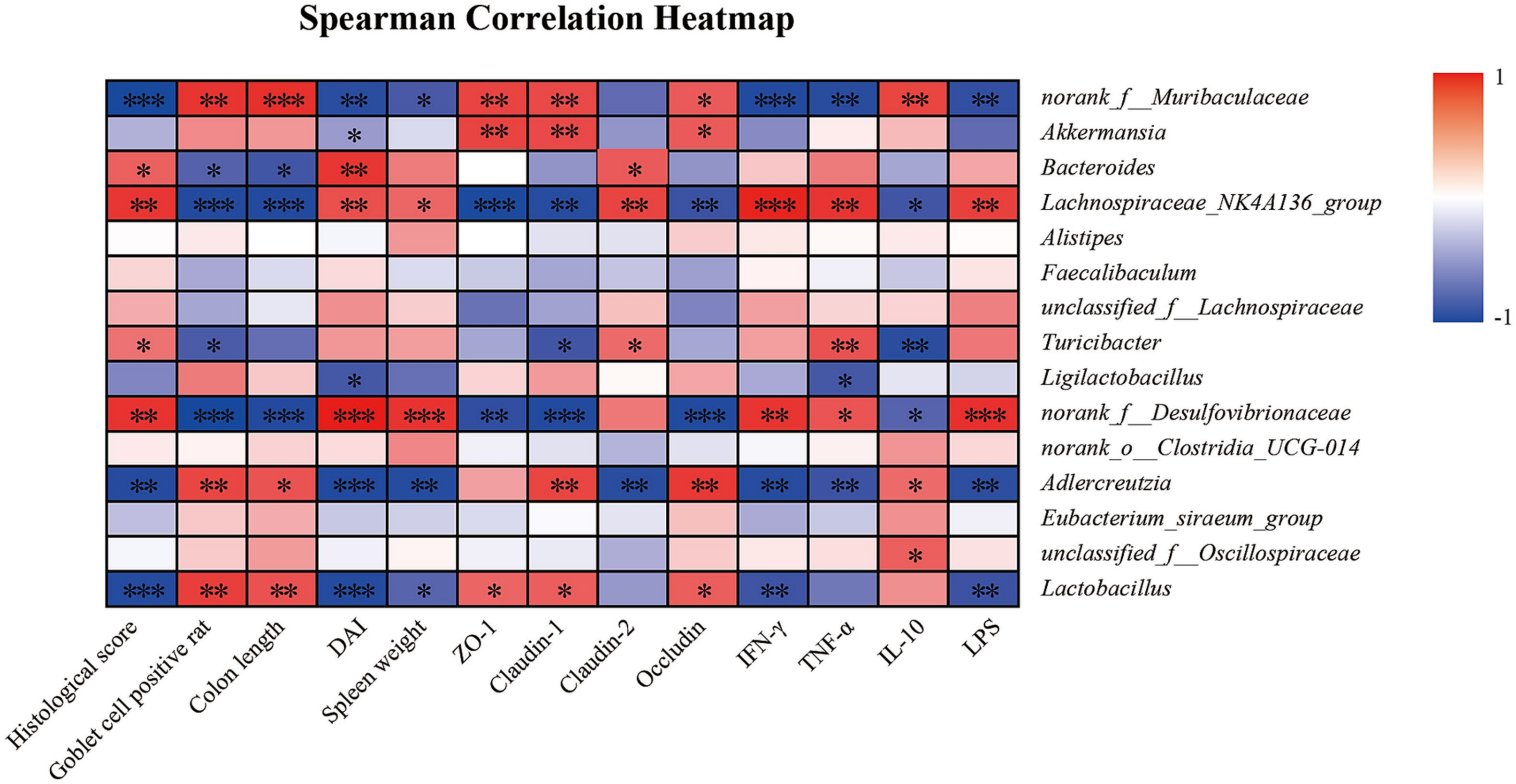
Correlation between key taxa and inflammation indices. *p < 0.05; **p < 0.01; ***p < 0.001.

To explore potential functional relationships between gut microbiota and host physiology, we conducted Spearman correlation analysis (Figure 7). Genera associated with intestinal homeostasis, such as *norank_f_Muribaculaceae, Akkermansia*, and *Lactobacillus,* were positively correlated with tight junction (*ZO-1, Claudin-1, Occludin*) and the anti-inflammatory cytokine IL-10, and negatively correlated with proinflammatory cytokines (TNF-α, IFN-γ) and serum LPS. In contrast, taxa enriched in inflammatory conditions (*Desulfovibrio, Lachnispiraceae_NK4A136_group*) displayed the opposite correlation pattern. These results suggest that the protective effects of Arg and BeArg on intestinal barrier integrity and inflammation may be mediated, at least in part, by modulation of gut microbiota composition.

### 3.8 Arg exerts preventive effects against colitis through modulation of the gut microbiota

**Figure 8:**
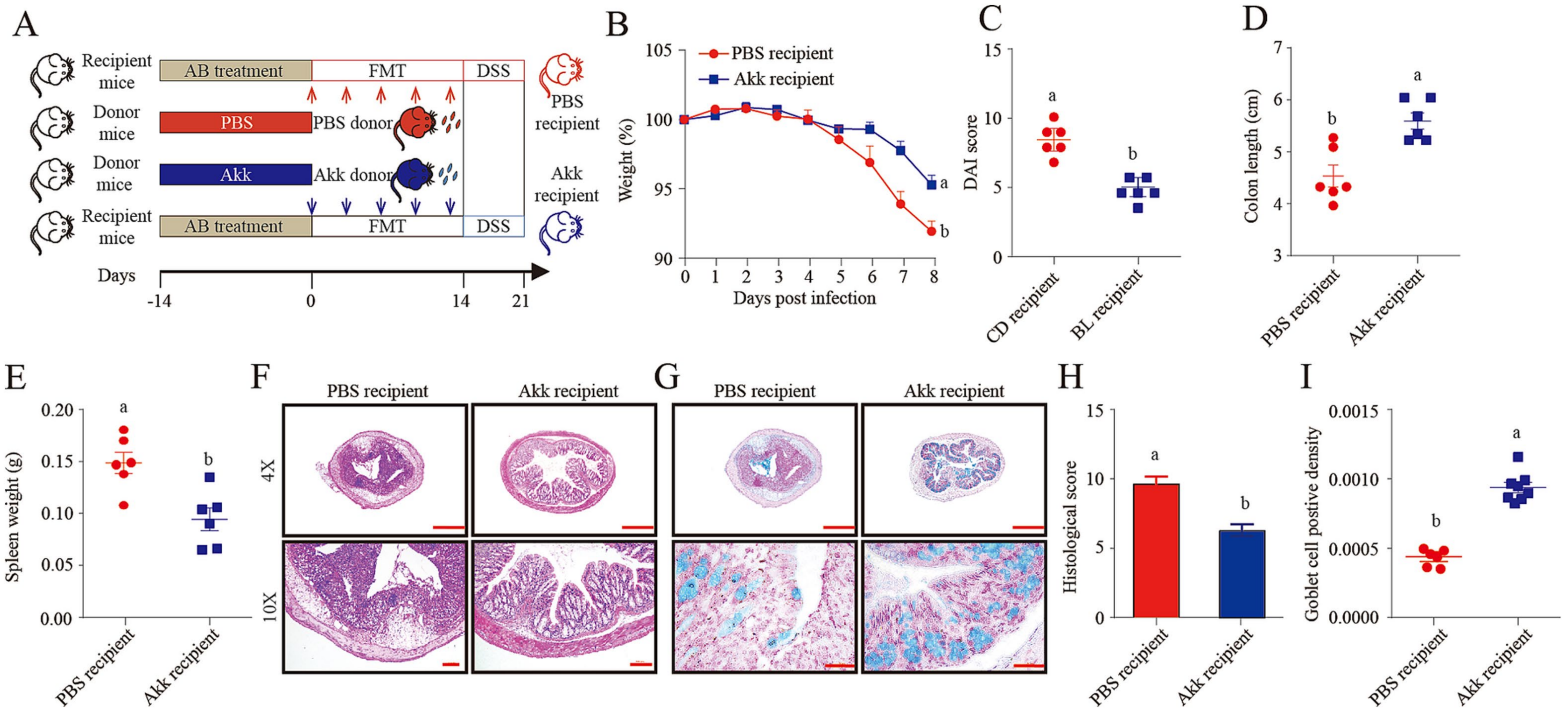
FMT confirms the preventive effects of Arg against colitis through gut microbiota modulation. **(A)** Experimental design; **(B)** Body weight loss; **(C)** DAI score; **(D)** Colon length; **(E)** Spleen weight. **(F)** Representative H&E-stained section images. **(G)** Representative AB stained section images. **(H)** Histological score; **(I)** Goblet cell positive density. *n* = 6. Groups labeled with different letters are significantly different (*p* < 0.05).

16S rRNA sequencing analysis showed that the gut microbiota profile of mice in the BeArg group was highly similar to that of the Arg intervention group, implying that the preventive effects of Arg on colitis may be mediated through regulation of the gut microbiota. To further validate this possibility, an FMT experiment was performed (Figure 8A). Recipient mice transplanted with fecal microbiota from Arg-treated donors showed significant protection against DSS-induced colitis, as reflected by reduced body weight loss and alleviated colon shortening (Figures 8B–E). In addition, H&E and AB staining further confirmed these protective effects at the histopathological level (Figures 8F–I). Taken together, these findings suggest that Arg exerts preventive effects on colitis, at least partly, by reshaping gut microbiota composition, which may help explain the similar protective outcomes observed in the BeArg and Arg treated groups.

## 4 Discussion

UC is a chronic, relapsing gastrointestinal disease and remains a substantial global health burden. Accumulating evidence indicates that diet-based strategies serve as a biologically plausible adjunct for colitis prevention and management. For instance, a long-term prospective studies have suggested that higher dietary fiber intake is associated with a lower risk of UC (18). L-glutamine supplementation in UC rats prevented hemorrhagic damage and improved oxidative stress markers (19). Dietary supplementation with *Lactobacillus acidophilus* could ameliorate UC by promoting Treg differentiation and inhibiting M1 macrophage polarization (20). Our previous study demonstrated that Arg supplementation could markedly alleviate colitis symptoms in murine models (17); however, it remains unclear whether dietary Arg exerts preventive or therapeutic effects. The present study, therefore, systematically compared prophylactic Arg supplementation before DSS exposure (BeArg) with therapeutic supplementation initiated during DSS administration (DuArg), and evaluated their effects on disease phenotype, histopathology, inflammation cytokines, barrier-related gene expression, and gut microbial composition.

Previous work reported that a 20 g/day oral dose of Arg produced no adverse effects in adults (21). Using standard human-animal body surface area conversion, the 2 g/(kg·bw) dose administered in this study is equivalent to 15.4 g for a 70-kg adult, which is below that observed safe level. A central finding of this study is that the timing of Arg exposure critically determined its protective efficacy. Preventive administration markedly attenuated DSS-induced body weight loss, lowered DAI score, and preserved colon length, whereas therapeutic administration produced only a modest benefit, primarily reflected by colon length (Figure 1). These data indicate that Arg may be more effective in limiting the initiation and early amplification of DSS-induced mucosal injury than in reversing already established inflammatory damage. The phenotypic benefit observed in the BeArg group was strongly supported by histopathological evidence. H&E staining demonstrated that BeArg administration markedly reduced inflammatory cell infiltration, epithelial erosion, and crypt destruction (Figures 2A,B). Consistently, AB staining revealed that preventive Arg administration effectively preserved goblet cell numbers and mucin production, indicating maintenance of the mucus layer. Given the critical role of the mucus barrier in limiting luminal antigen exposure and bacterial translocation (22, 23), preservation of goblet cells likely contributed to reduced inflammatory burden and improved clinical outcomes. In contrast, therapeutic Arg administration led to only partial histological improvement, with persistent epithelial disruption and reduced mucin staining, consistent with its limited efficacy in improving clinical phenotype (Figures 2C,D).

Increased epithelial permeability is the well-recognized hallmark of UC (24). DSS-induced downregulation of *ZO-1*, *Claudin-1*, and *Occludin,* together with increased *Claudin-2,* is a classic signature of epithelial leakiness in experimental colitis. In the present study, BeArg partially normalized this profile and markedly reduced circulating LPS. By comparison, the DuArg administration had limited effects on the expression of these tight junction proteins, in line with its weaker impact on disease phenotype (Figures 1, 3). Importantly, these barrier-associated improvements aligned with a coordinated shift in the mucosal cytokine milieu. Compared with the DSS group, both Arg and BeArg significantly decreased TNF-α and IFN-γ at the mRNA and tissue protein levels, while increasing IL-10 (Figure 4). This cytokine pattern is mechanistically coherent. TNF-α and IFN-γ are well-established mediators of epithelial barrier dysfunction and can cooperatively disrupt tight-junction organization, increase permeability, and perpetuate mucosal injury (25, 26). Conversely, IL-10 is a key counter-regulatory cytokine required for maintaining intestinal immune homeostasis, and its enhancement is consistent with a pro-resolution shift (27). By contrast, DuArg produced only a non-significant downward trend in TNF-α and IFN-γ, paralleling its limited effects on tight-junction gene expression and supporting the notion that treatment window influences the capacity to reprogram established inflammatory circuits.

The microbiome data dovetail with the barrier and inflammation phenotypes observed earlier. DSS induced a pronounced dysbiosis marked by reduced α-diversity and clear separation in *β*-diversity space, consistent with a loss of ecological resilience that can sustain epithelial injury and mucosal inflammation (28). At the phylum level, the DSS-associated decrease in Bacteroidota and expansion of taxa linked to sulfur metabolism/anaerobic inflammation-related niches has been described across multiple studies (29). In the present results, Arg and BeArg moved these phylum-level patterns toward the CON group, whereas DuArg exhibited only modest modulation (Figure 5; Supplementary Figure S1).

Genus-level changes provide a more specific biological context. DSS reduced *Akkermansia*, while Arg and BeArg increased its relative abundance. This direction is consistent with recent DSS studies in which *Akkermansia muciniphila* administration is associated with improved colitis phenotypes and recovery of the intestinal barrier (30, 31). In parallel, LEfSe enrichment of *Muribaculaceae*-related signals in control-associated profiles aligns with recent syntheses noting that *Muribaculaceae* abundance often declines in IBD models and correlates with mucosal-layer features (Supplementary Figures S2, S3) (32). The Arg-group enrichment of *Odoribacter* is also notable; recent work in UC-relevant contexts has highlighted *Odoribacter*-linked bile acid patterns and associations with reduced inflammatory activity, supporting its frequent categorization as a remission-associated taxon in some datasets (33, 34). Conversely, DSS increased several taxa commonly reported in rodent colitis-associated dysbiosis, including *Desulfovibrionales* and *Helicobacter*. Recent experimental studies show that *Desulfovibrio vulgaris* expansion or colonization is associated with more severe DSS colitis and compromised barrier function (35). *Helicobacter* colonization has likewise been linked to barrier disruption and tight-junction impairment *in vivo* and in epithelial models (36). Notably, *Lachnospiraceae_NK4A136_group* is often discussed as reduced in IBD patient microbiomes in some cross-sectional summaries, but its directionality can vary by host background, disease stage, diet, and analytical resolution (37). Therefore, the DSS-associated enrichment observed here is not necessarily contradictory; rather, it highlights the context-dependence of genus-level signatures and the need to avoid overgeneralizing “beneficial *vs* harmful” labels.

Correlation analysis further suggested a close association between gut microbial composition and inflammatory responses (Figure 7). In addition, 16S rRNA sequencing analysis revealed that the gut microbiota profile of mice in the BeArg group closely resembled that of the Arg intervention group, indicating that early Arg supplementation may establish an intestinal homeostasis microbial environment before DSS exposure. To further demonstrate that Arg exerts a preventative effect against colitis by remodeling the gut microbiota, we conducted an FMT experiment (Figure 8A). The results showed that recipient mice transplanted with fecal microbiota from Arg-treated donors were less susceptible to DSS-induced colitis, as evidenced by attenuated body weight loss, reduced colon shortening, and improved histopathological injury. Accumulating evidence indicates that dysbiosis is closely linked to impaired epithelial barrier integrity, exaggerated mucosal immune activation, and the progression of colitis, highlighting the gut microbiota as a central regulator of intestinal homeostasis (38). These findings indicate that the better efficacy observed with BeArg than with DuArg may be attributed, at least in part, to the ability of BeArg to reshape gut microbiota composition prior to DSS challenge, thereby promoting intestinal homeostasis and enhancing resistance to subsequent inflammatory injury.

Taken together, the main contribution of the present study is not simply that Arg improves DSS colitis, but that the timing of administration critically influences its efficacy. Although the present study has demonstrated that Arg may be more effective as a prophylactic modulator of mucosal susceptibility than as a stand-alone intervention for established acute colitis, several limitations should be acknowledged. Arg is a known substrate for NO synthesis, which could modulate intestinal immune responses and barrier function, and influence NF-κB signaling (39, 40). Based on these well-established pathways, it is plausible that Arg-mediated NO production contributes to suppression of NF-κB activation and attenuation of intestinal inflammation. Future experiments can focus on these pathways to directly evaluate their involvement, which will provide clearer evidence for the protective effects of Arg in colitis. Additionally, the assessment of intestinal barrier integrity in this study was based solely on mRNA expression of tight junction proteins. mRNA levels may not fully reflect protein abundance or proper localization at the membrane. Although serum LPS was measured as a functional readout, future studies will include protein-level analyses to more directly evaluate barrier integrity. Finally, a limitation of the current study is the exclusive use of male mice. Mounting evidence highlights that gut microbiota composition, local mucosal immunity, and responses to microbiota-targeted interventions are profoundly sex-dependent (41, 42). Future studies incorporating both male and female cohorts will be crucial for comprehensively assessing the beneficial effects of Arg.

## 5 Conclusion

**Figure 9:**
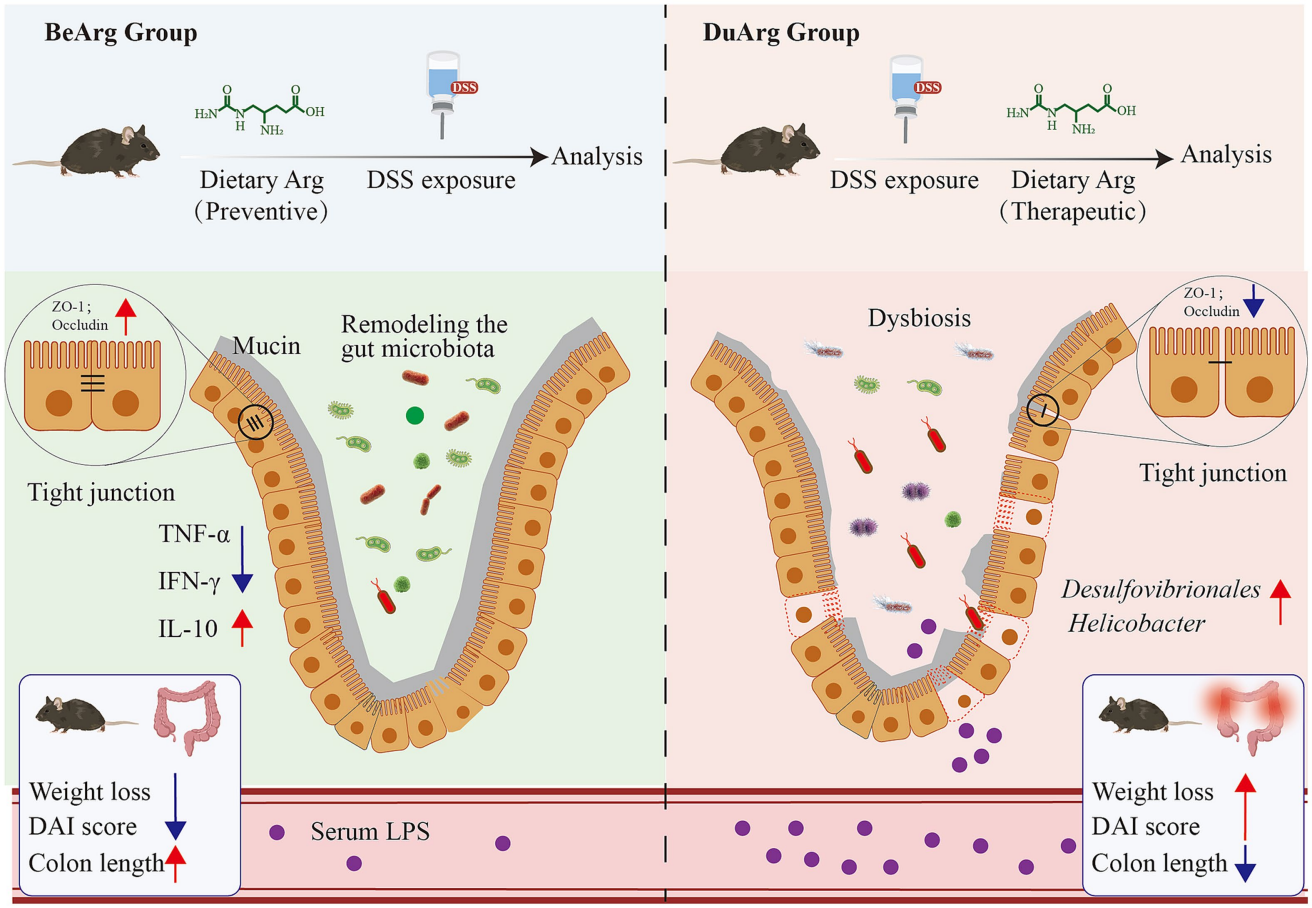
Schematic representation of the research.

In conclusion, dietary Arg supplementation alleviates DSS-induced colitis, and its preventive administration is more effective than therapeutic intervention. The superior efficacy of early Arg supplementation may be attributed, at least in part, to its ability to remodel the gut microbiota before inflammatory challenge, thereby preserving intestinal homeostasis and enhancing resistance to colitis (Figure 9). This study highlights intervention timing as a critical determinant of Arg efficacy and supports Arg as a potential prophylactic nutritional strategy for colitis prevention.

## Data Availability

The raw data generated in this study can be found in the NCBI Sequence Read Archive (SRA) under BioProject accession number PRJNA1439605.
