# Supplementary material for: Dietary L-arginine supplementation exerts preventive effects on colitis through modulation of the gut microbiota
Source: Front Nutr. 2026 May 29;13:1848380. doi: 10.3389/fnut.2026.1848380 (PMC13260480; doi:10.3389/fnut.2026.1848380)
Supplement: Supplementary file 1 [file Supplementary_file_1.DOCX]

Dietary L-Arginine supplementation exerts preventive effects on colitis through modulation of the gut microbiota

Lerun Gong ^†^, Jing Kong ^†^, Xiaoying Shao, Weiming Meng, Ruxin Zhang, Yanxi Zhang, Yu Feng^^[[1]](#footnote-1)^*^

**This file includes:**

Fig. S1. Effects of BeArg and DuArg interventions on the composition of the mouse gut microbiota;

Fig. S2. LEfSe analysis identifying differential gut microbial taxa among experimental groups;

Fig. S3. Rank-sum test of genus-level differences between two groups.;

Table S1. Disease activity index;

Table S2. Histopathological scores;

Table S3. Primer sequences.

**Fig. S1**

| 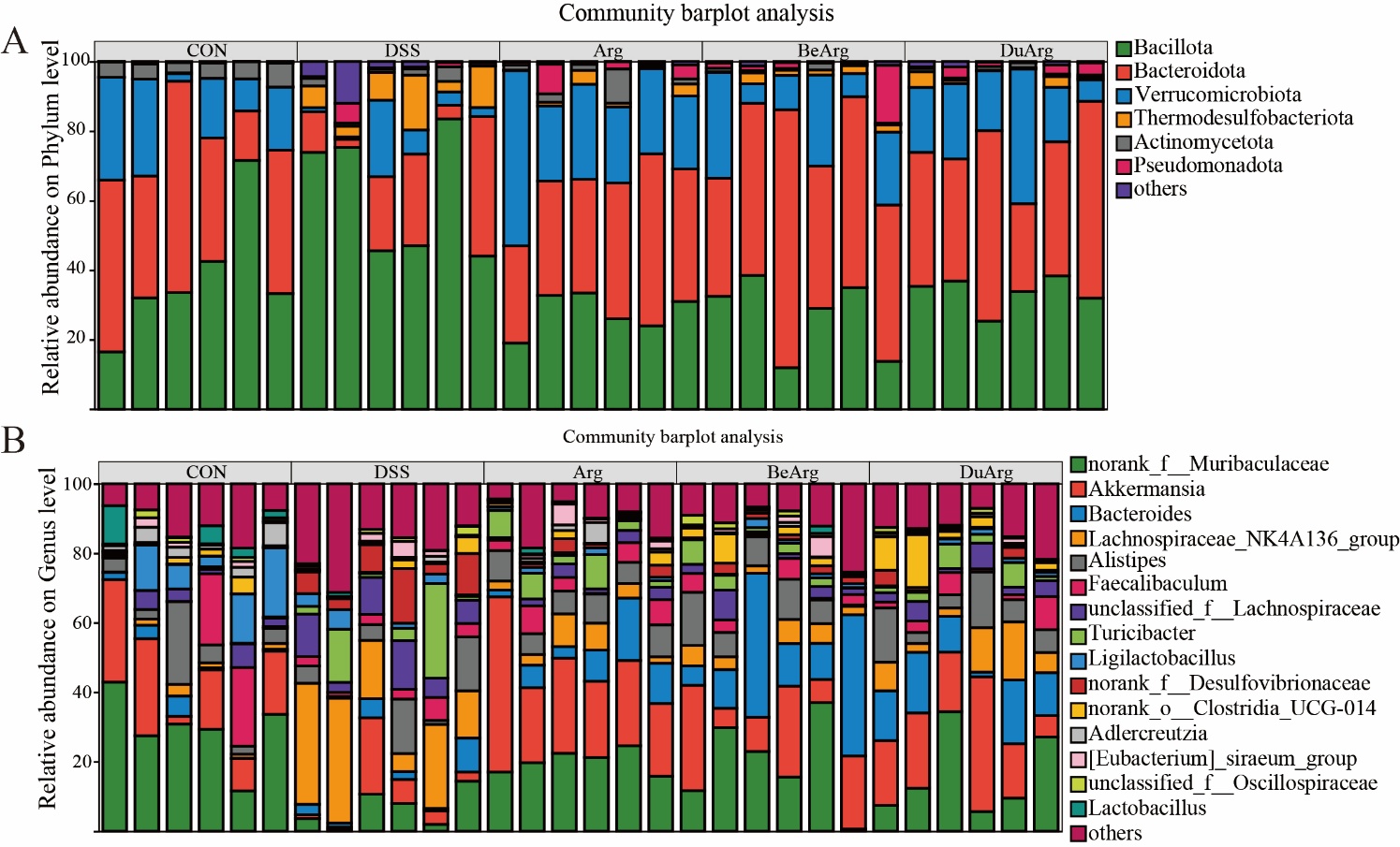 |  |
| --- | --- |
|  |  |

**Fig. S1 Effects of BeArg and DuArg interventions on the composition of the mouse gut microbiota.** (A: Relative abundance of gut microbiota at the phylum level; B: Relative abundance of gut microbiota at the genus level)

**Fig. S2**

**
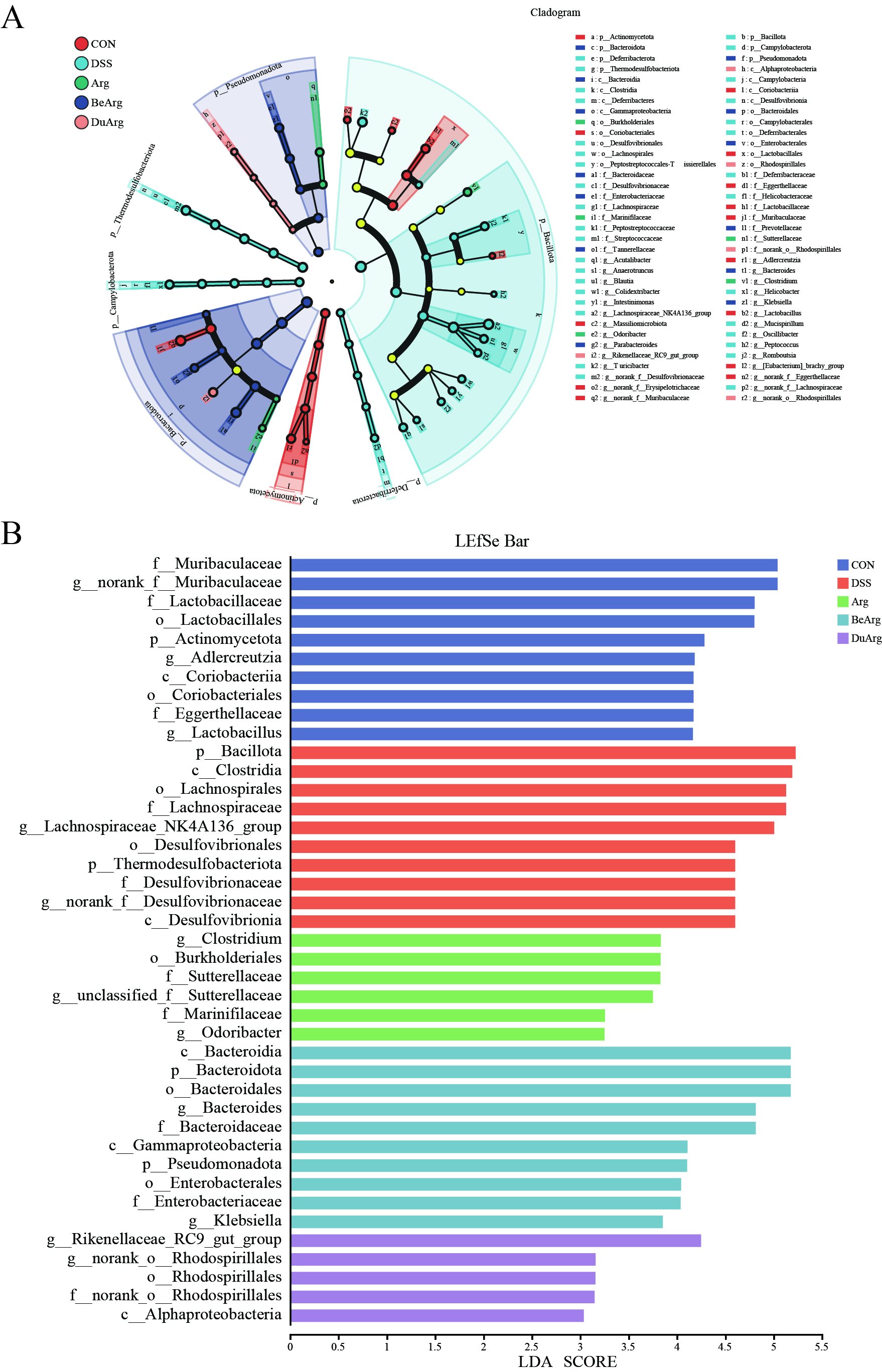
**

**Fig. S2 LEfSe analysis identifying differential gut microbial taxa among experimental groups.** (A: Cladogram generated by LEfSe showing taxa significantly enriched in each group; B: LDA score plot displaying the effect size of discriminative taxa across groups)

**Fig. S3**

| 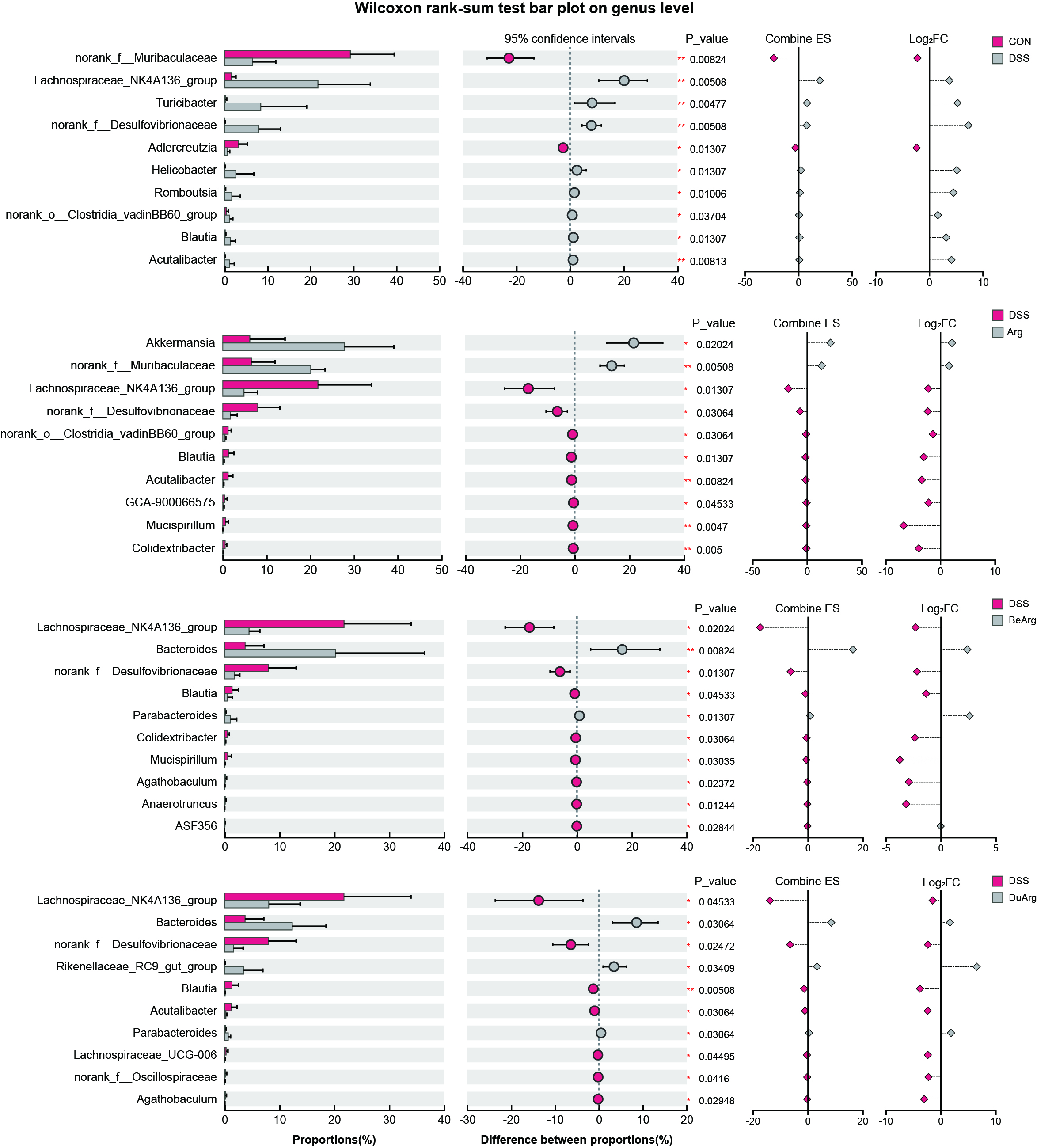 |
| --- |

**Fig. S3 Rank-sum test of genus-level differences between two groups.** Genus-level relative abundances were compared between the two groups using a rank-sum test. Bars indicate group means, and dots represent individual animals. P values are shown in the figure, with P < 0.05 considered statistically significant.

**Table S1**

**Table S1.** Disease activity index scores

| Score | Weight loss（%） | stool consistency | general health |
| --- | --- | --- | --- |
| 0 | 0 | Normal | Normal |
| 1 | 1-5 |  | Mild |
| 2 | 6-10 | Loose | Moderate |
| 3 | 11-15 |  | Severe |
| 4 | >16 | Diarrhea | Moribund |

**Table S2**

**Table S2.** Histopathological scores

| Score | Inflammation | Epithelial hyperplasia | Erosion and ulceration | Extent of lesion |
| --- | --- | --- | --- | --- |
| 0 | absent | None | None | 0 |
| 1 | minimal in the mucosa | mild | rare erosions | <10 |
| 2 | mild affecting mucosa  and sub-mucosa | mild with minimum goblet cell loss | some erosions | 10-25 |
| 3 | moderate affecting mucosa, sub-mucosa, and sometimes transmural | moderate with mild goblet cell loss | multiple erosions and ulcerations  with cryptitis and crypt abscesses | 25-50 |
| 4 | severe: often transmural | marked with moderate  to marked goblet cell loss | ulcers associated with necrosis and fibrosis | >50 |

**Table S3**

**Table S3.** Primer sequences

| Gene | Primer sequence |
| --- | --- |
| *GAPDH-F* | GTGTTCCTACCCCCAATGTGT |
| *GAPDH-R* | ATTGTCATACCAGGAAATGAGCTT |
| *Occludin-F* | TTGAAAGTCCACCTCCTTACAGA |
| *Occludin-R* | CCGGATAAAAAGAGTACGCTGG |
| *Claudin1-F* | AGGTCTGGCGACATTAGTGG |
| *Claudin1-R* | CGTGGTGTTGGGTAAGAGGT |
| *Claudin2-F* | AGTGGCTGTAGTGGGTGGAG |
| *Claudin2-R* | AAAGGATGACTCCGGCTACC |
| *ZO1-F* | GATCCCTGTAAGTCACCCAGA |
| *ZO1-R* | CTCCCTGCTTGCACTCCTATC |
| *TNFa-F* | TGATCGGTCCCCAAAGGGAT |
| *TNFa-R* | TGTCTTTGAGATCCATGCCGT |
| *IFNg-F* | CAGCAACAGCAAGGCGAAAAAGG |
| *IFNg-R* | TTTCCGCTTCCTGAGGCTGGAT |
| *IL10-F* | CGGGAAGACAATAACTGCACCC |
| *IL10-R* | CGGTTAGCAGTATGTTGTCCAGC |

1. **Correspondence:** School of Life Science and Engineering, Jining University, Jining, 273155, China.;

   **E-mail:** fengyu9459@163.com [↑](#footnote-ref-1)
